# Utilization of lymph node elastography in the axillary staging of breast cancer

**DOI:** 10.3389/fonc.2025.1478701

**Published:** 2025-03-13

**Authors:** Marek Smolar, Eva Kudelova, Ivana Danova, Vincent Lucansky, Zuzana Dankova, Diana Musova, Marian Grendar, Lenka Nosakova, Peter Uhrik, Marek Samec

**Affiliations:** ^1^ Clinic of General, Visceral and Transplant Surgery, Jessenius Faculty of Medicine, Comenius University in Bratislava, University Hospital Martin, Martin, Slovakia; ^2^ Department of Pathological Physiology, Jessenius Faculty of Medicine in Martin, Comenius University in Bratislava, Martin, Slovakia; ^3^ Biobank for Cancer and Rare Diseases, Jessenius Faculty of Medicine in Martin (JFMED CU), Comenius University in Bratislava, Martin, Slovakia; ^4^ Biomedical Centre Martin, Jessenius Faculty of Medicine in Martin (JFMED CU), Comenius University in Bratislava, Martin, Slovakia; ^5^ Clinic of Gastroenterological Internal Medicine, Jessenius Faculty of Medicine, Comenius University in Bratislava, University Hospital, Martin, Slovakia; ^6^ Department of Medical Biology, Jessenius Faculty of Medicine in Martin, Comenius University in Bratislava, Martin, Slovakia

**Keywords:** elastography, breast cancer, lymph node, ultrasonography, metastasis, imaging techniques

## Abstract

**Background:**

The differential diagnosis of lymphadenopathy is an important determinant of prognosis in patients with breast cancer (BC). Invasive, fine needle aspiration (FNA) biopsy has been long considered as the gold standard for differentiating malignant lymph nodes (LN) from benign ones. Ultrasonography (USG) evaluation is a useful, rapid, and user-friendly imaging tool for LN assessment due to its high resolution. Compared to USG, ultrasound elastography is a relatively novel non-invasive method to differentiate benign and malignant lesions based on the stiffness heterogeneity of the tissue. The purpose of our study was to compare non-invasive imaging techniques, conventional USG, and strain elastography, to differentiate benign and malignant LNs lesions in a cohort of patients with early BC.

**Methods:**

In total, 50 patients (48 women and 2 men) with histologically confirmed early BC were evaluated by conventional USG in B-mode followed by strain elastography (using parameters: pattern, strain ratio, hue histogram) for assessment of axillary LNs status. The surgical treatment included surgery of regional LNs (sentinel LN biopsy or axillary dissection), which served as the gold standard in statistical processing.

**Results:**

The USG B-mode was found to have a sensitivity of 68.75% and a specificity of 61.54%. Among strain elastography parameters, the elastographic pattern showed the highest specificity (66.67%) while the sensitivity was 83.3%. The strain ratio showed 100% sensitivity and 55.6% specificity, followed by a hue histogram with a sensitivity of 72.2%, but specificity was only 25.9%.

**Conclusion:**

Despite promising data, monitored parameters currently cannot reliably replace sentinel LN biopsy. However, the monitored parameters represent an appropriate additional tool that can be used to refine preoperative staging, better targeting of FNA biopsy, and more accurate assessment of LNs in follow-up patients within the dispensary.

## 1 Introduction

Breast cancer (BC) is the most commonly diagnosed cancer worldwide since 2022, with an estimated 2.29 million new cases a year (11,7% of all cases), and it is the fifth most common cause of death associated with oncologic disease, with 665 000 deaths (6,9% of all cases). Considering women alone, it has not only the highest incidence (24,5% of all cases) but also it is a leading cause of cancer-related mortality (15,5% of all cases) (1, 2). In addition, the incidence of BC in Europe has increased rapidly in recent decades, and it is expected to increase in the future (2, 3).

The selection of standard treatment for BC depends on subtype and metastatic status. The surgery, most often in combination with chemo- or radiotherapy, is the most conventional approach to disease management (4). Both a total mastectomy or lumpectomy, assuming that clear margins can be achieved, have been shown consistently to be equivalent with regard to relapse-free and overall survival (5). More than 90% of BC are not metastatic at the time of diagnosis; thus, preventing recurrence after therapeutic tumor eradication is highly important (4). Lymph node (LN) removal serves both a diagnostic purpose (determining the anatomic extent of BC) and a therapeutic purpose (removal of cancerous cells) (5). However, due to the high morbidity caused by BC-related lymphedema (BCRL) (6), an alternative, less radical approach enabling the surveillance without the risk of such profound consequences is being applied whenever it is feasible. Axillary lymph node dissection (ALND) still remains the standard of care in any patient with clinically evident axillary involvement at diagnosis who undergoes surgery as initial treatment (7).

Sentinel lymph node (SLN) surgery provides a certain reduction of the side effects of lymph-node surgery but still offers outcomes equivalent to ALND. Clinical trials demonstrated that in women with clinically node-negative BC, there was no significant difference in recurrence nor survival outcomes between women who underwent full ALND vs. women who underwent SLN biopsy, with conversion to ALND only if the SLN was positive (7).

Axillary LN sampling by ultrasound-guided fine needle aspiration (US-FNA) has been lately advocated as a less invasive in comparison to SLN, however; the results of the study of Attieh et al. suggested that FNA is not a reliable tool in triaging patients in need for ALND and leads to overtreatment of 43% patients when positive while depriving a significant percentage (16.7%) of patients from necessary therapy when negative (8). In addition to the frequent inconclusive results of FNA and the fact that it often utilizes radioactive trackers for visualization (9, 10), it is still an invasive method. Thus, it is important to consider the risk/benefit ratio to patients.

Truly non-invasive, very common, and accessible palpation diagnostic is unreliable and subject to the individual experience of the examining physician (11). Several non-invasive imaging methods like mammography (MM), ultrasonography (USG), magnetic resonance imaging (MRI), and computed tomography (CT) routinely used in BC diagnostic have been shown to be superior to palpation examination (12, 13). One of such non-invasive imaging technique used for the axillary staging of BC is ultrasound elastography. Elastography visualizes different deformation degrees of tissues with different hardness coefficients after being compressed by external forces. It can assess the deformability of tissues, show tissue elasticity, and reflect the biological characteristics of lesions. It is capable of detecting lesions that cannot be detected by routine US (14, 15). Currently, the most commonly used types of elastography are strain elastography (SE) and shear wave elastography (SWE) (16). SE involves applying force through probe pressure or natural mechanical forces, such as carotid pulsation. On the other hand, SWE uses an imaging system to induce a shear wave in the tissue. In both methods, the tissue’s response to these mechanical stimuli helps estimate its mechanical properties (17)

The objective of this study was to assess the sensitivity and specificity of the LN elastography method (SE approach), to determine the most suitable parameters for estimation of the lymphatic nodes infiltration by metastases, and to identify the relationships of output data to individual parameters characterizing BC.

## 2 Materials and methods

This study was conducted in accordance with the Declaration of Helsinki and approved by the institutional ethical committee of Jessenius University of Medicine in Martin (*EK 32/2020).* Informed written consent was obtained from each patient included in the study.

### 2.1 Patients

This study enrolled 50 patients (48 women and 2 men) hospitalized at the Surgical Clinic of the Jessenius University of Medicine in Martin and University Hospital in Martin in 2020 – 2021 with histologically confirmed BC. The age range of all patients was 59 ± 13 years. Patients with early-stage breast tumors, specifically DCIS (Ductal Carcinoma *In Situ*) and T1-T2 stages, were included in the study (they were determined by mammography and ultrasonography - USG). The characteristics of these tumors are now detailed based on both their molecular-biological subtypes (Luminal A, Luminal B, HER2 type, Triple-negative) and their size, classified according to the 8th edition of the TNM system. Tumor sizes range from Tis (DCIS without basement membrane invasion), to T1a (tumors up to 5 mm), T1b (up to 10 mm), T1c (up to 19 mm), and T2 (tumors between 2 and 5 cm). These criteria were adopted following the American Joint Committee on Cancer’s Staging System for Breast Cancer, Eighth Edition, which offers a standardized framework for the categorization of breast cancer stages and helps ensure consistency with other clinical studies (reference: http://www.breastsurgeonsweb.com/wp-content/uploads/downloads/2020/10/AJCC-Breast-Cancer-Staging-System.pdf), All patients underwent standard biopsy of sentinel LN or axillary dissection. Histopathological results were subsequently used as reference standards for comparison of USG and elastography examination. Sentinel LN detection was performed using radioactive colloids with the 1-day protocol or 2-day protocol. Patent blue dye was used in one case (**Table 1**).

**Table 1:** Methods of sentinel LN detection.

Sentinel LN detection	n (%)
Radionuclide method	47 (94%)
1-day protocol	7 (14%)
2-day protocol	40 (80%)
Patent blue dye	1 (2%)

### 2.2 Evaluated parameters

Clinical, USG, and elastographic parameters were monitored for each patient involved in the study. All evaluated parameters were correlated with histological analysis (the gold standard for detecting LNs involvement in malignancy). Also, sensitivity, specificity, positive predictive value (PPV), and negative predictive value (NPV) were determined.

#### 2.2.1 Clinical and conventional USG parameters

Clinical parameters involved LN staging of BC (cN0 = no palpable or visibly enlarged inguinal LN, cN1 = palpable mobile unilateral inguinal LN). Additionally, USG parameters included spherical LN, change in echogenicity – the entire LN is hypoechoic, and signs of a ruptured capsule.

#### 2.2.2 Elastography parameters

The strain elastography method was utilized for the examination of patients. A certified doctor for an elastographic examination performed the measurement using a Hitachi USG device. The preoperative measurement was realized the day before surgery or in the morning on the day of the surgery. The monitored parameters included B-mode examination, strain ratio (SR), hue (strain) histogram (SH), and pattern. According to the protocol, three LNs were examined. To reduce measurement errors, SR and SH were realized in triplicates.

### 2.3 Statistical analysis of the data

Data analysis was performed in the R environment (R Foundation for Statistical Computing, Vienna, Austria) version 4.0.5. Discrete data were summarized using frequencies and relative frequencies (displayed in tables). Pivot tables were displayed using a mosaic chart. Continuous variables were summarized using mean and standard deviation. The null hypothesis of no association between two factors was tested by the Fisher test and the absence of a trend in the contingency table was assessed by the Cochran Armitage test. Welch’s t-test was used to test the null hypothesis of equality of population means in two populations. Data normality was assessed using a quantile-quantile plot with a 95% confidence band constructed using the bootstrap method. The empirical ROC curve was used to assess the discriminative ability of the predictors. Youden’s index was taken as the optimal point on the ROC curve (determining the sensitivity and specificity of the investigation methodology).

## 3 Results

### 3.1 Tumors characteristics

Based on preoperative biopsy and histopathology analysis, the most frequent tumors in our patients cohort were invasive breast carcinoma no special type (NST) (72%), followed by invasive lobular carcinoma (14%), invasive breast carcinoma with apocrine differentiation (4%), ductal carcinoma *in situ* (DCIS) (4%), mucinous carcinoma (4%) and micro-invasive carcinoma (4%). According to molecular classification, tumors were divided into luminal A (56%), luminal B (24%), HER2 (4%), and triple-negative BC (12%). Clinical examination of tumor size using imaging methods revealed a higher prevalence of patients with T1c tumor (50%), followed by T2 (24%), T1b (22%), T1a (2%), and Tis (2%). According to USG and clinical signs of nodal status, two patients underwent axillary dissection. Patients and tumor characteristics are presented in **Table 2**.

**Table 2:** Patients and tumor characteristics.

Characteristics	n (%)
**Age (y)**	59 *± 13*
Gender	
Men	2 (4%)
Women	48 (96%)
Histopathology	
Invasive breast carcinoma NST	36 (72%)
Lobular invasive carcinoma	7 (14%)
Invasive breast carcinoma with apocrine differentiation	2 (4%)
DCIS	2 (4%)
Mucinous carcinoma	2 (4%)
Microinvasive carcinoma	1 (2%)
Molecular subtype	
Luminal A	28 (56%)
Luminal B	12 (24%)
HER2	4 (8%)
Triple-negative BC	6 (12%)
Clinical evaluation of tumor size	
T1c tumor	25 (50%)
T2	12 (24%)
T1b	11 (22%)
T1a	1 (2%)
Tis	1 (2%)
No. of sentinel LN removed	
1	1 (2%)
2	21 (42%)
3	23 (46%)
4	3 (6%)
Axillary dissection	2 (4%)

BC, breast cancer; DCIS, ductal carcinoma *in situ*; LN, lymph node; NST, no special type.

### 3.2 Clinical examination

**Figure 1:**
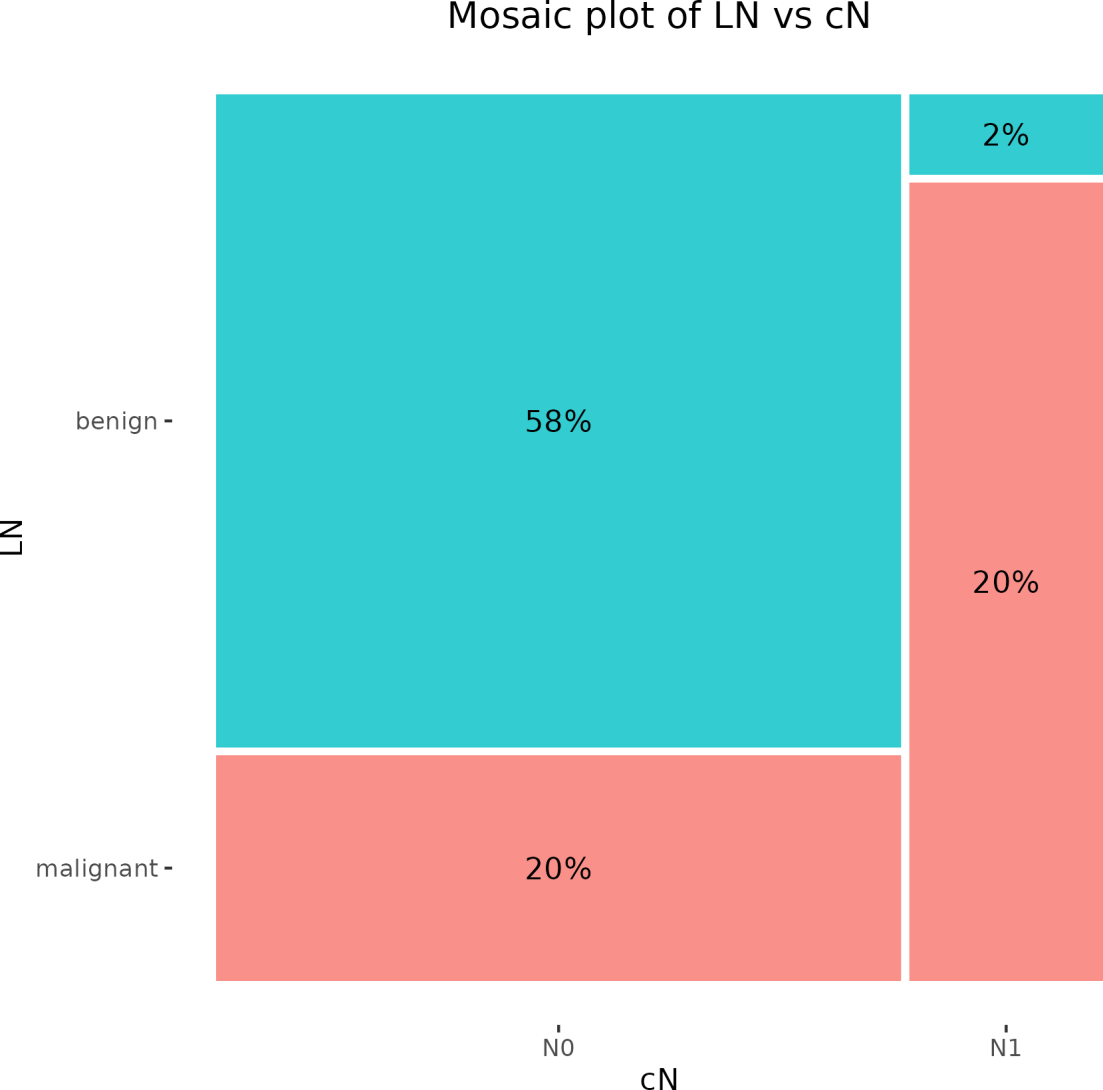
Mosaic plot illustrating the cross-tabulation of LN versus cN. The percentage displayed within each block represents the proportion of the total number of patients. LN, lymph node.

The clinical examination of regional LNs by palpation revealed 11 patients (22%) with cN1. On the other hand, 39 patients (78%) were classified as cN0. Subsequent histological validation confirmed one false positive result and ten false negative observations. The clinical assessment of LNs involvement in BC reached 50% sensitivity, 96.7% specificity, 90.9% PPV, and 74.4% NPV (**Figure 1**).

### 3.3 USG examination

**Figure 2:**
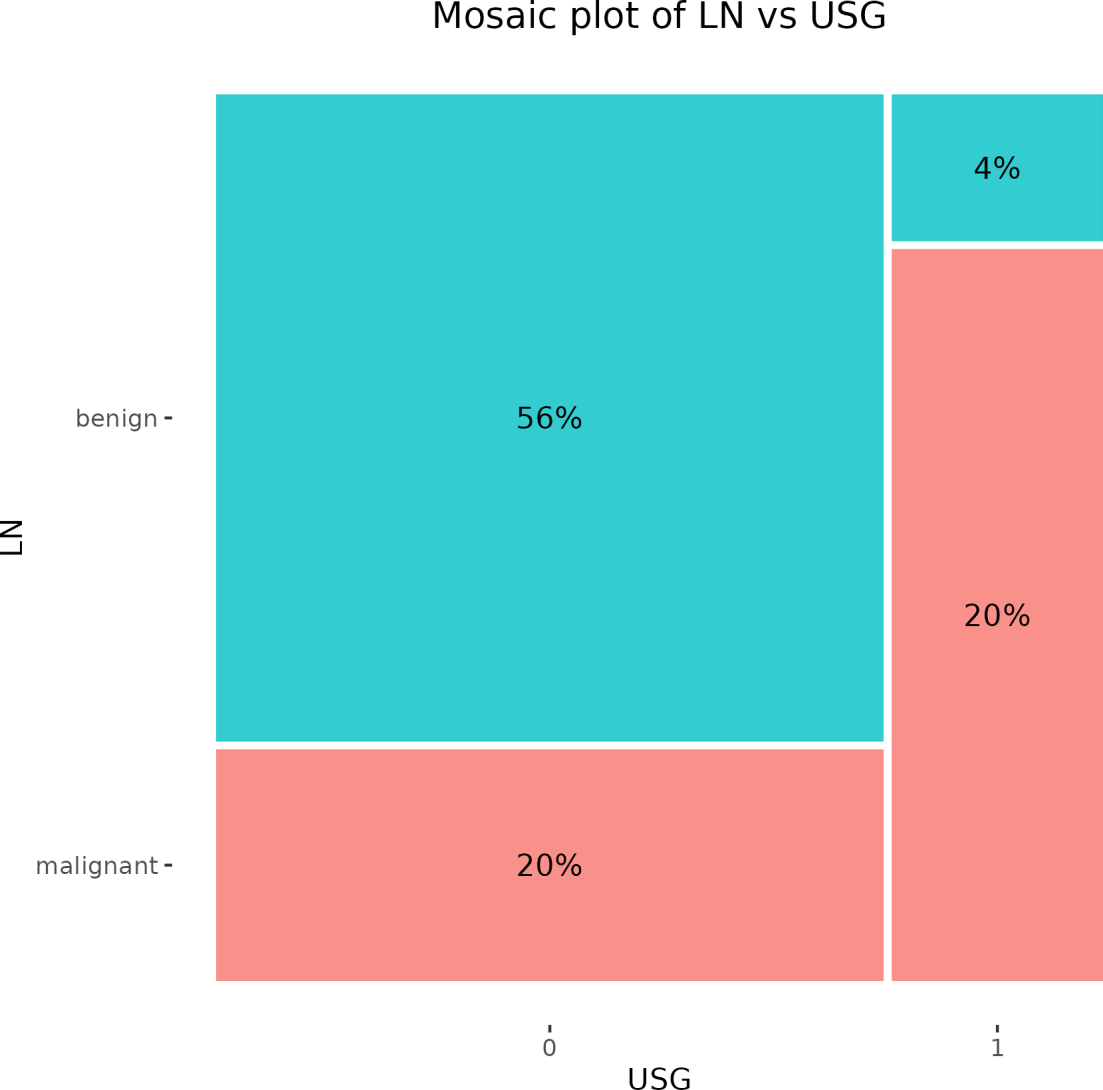
Mosaic plot illustrating the cross-tabulation of LN versus USG. The percentage displayed within each block represents the proportion of the total number of patients. LN, lymph node; USG, ultrasonography.

A preoperative USG examination of axillary LNs recognized false positive results in 2 cases, while the incidence of false positivity was documented in 10 cases. The PPV of this examination reached 83.3% and the NPV was 73.7% (**Figure 2**).

### 3.4 Elastography parameters

**Figure 3:**
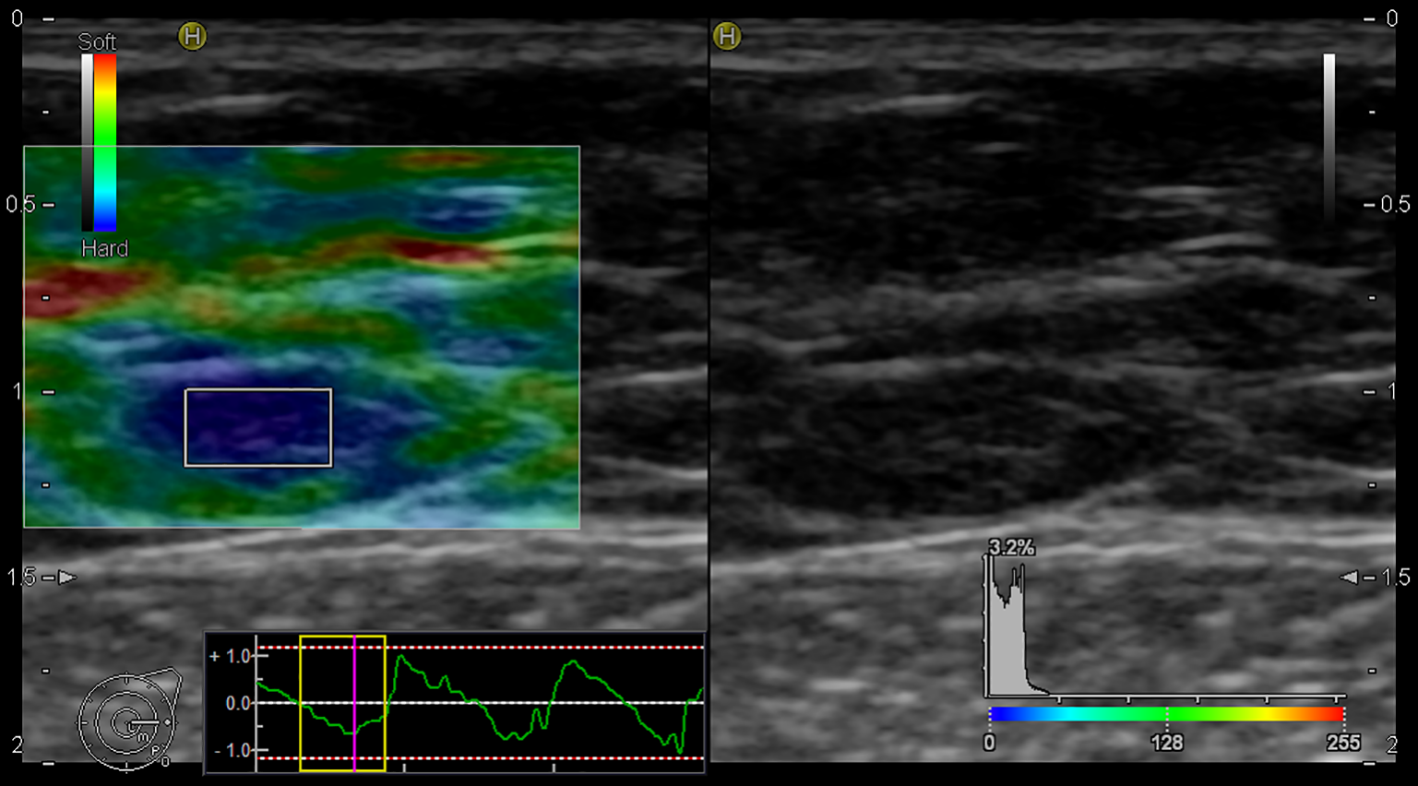
Elastography image of the metastatic lymph node - EG Pattern 5 (Tsukuba Elasticity Score 5).

**Figure 4:**
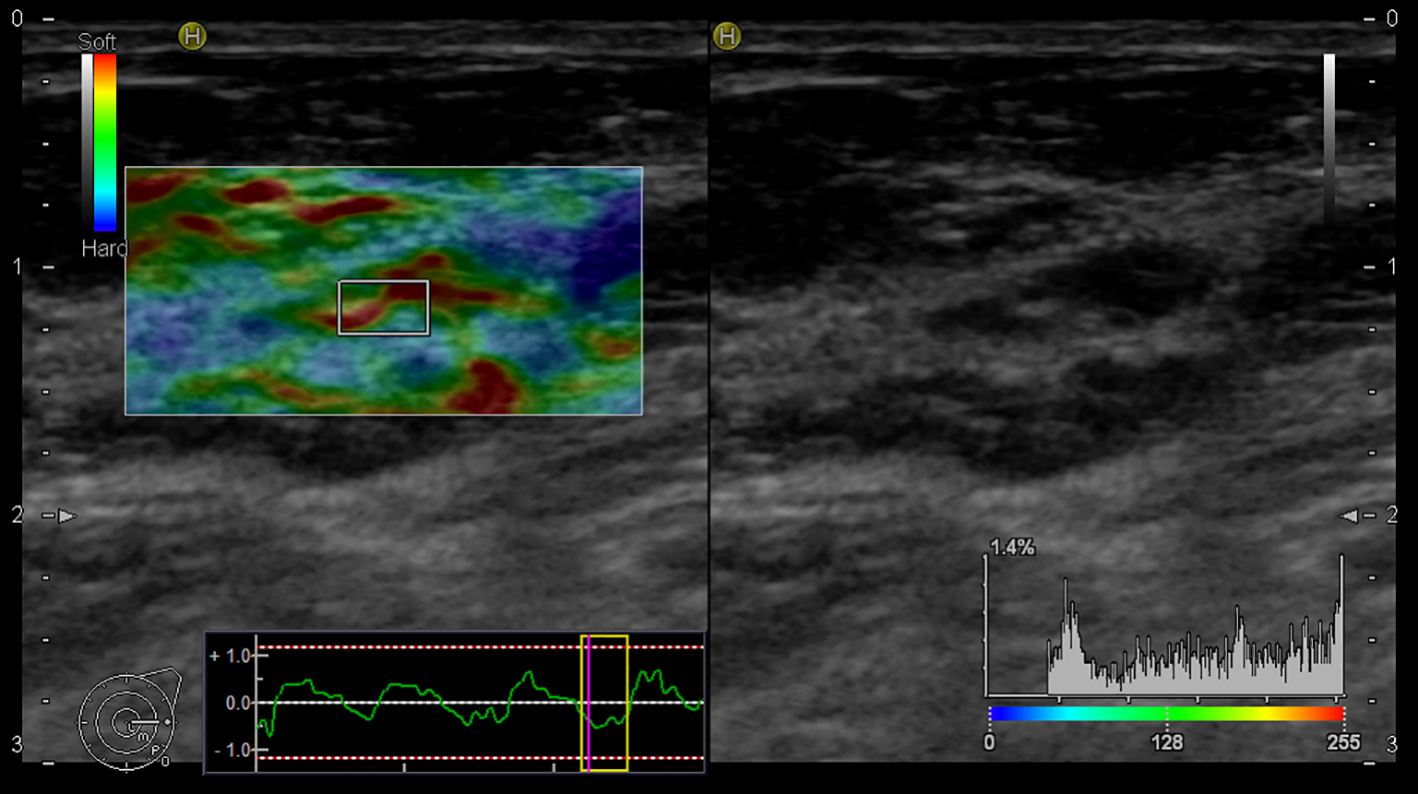
Elastography image of the benign lymph node - EG Pattern 2 (Tsukuba Elasticity Score 2).

In our study, the using of elastography for the assessment of LNs began with the evaluation of B-mode, followed by additional elastographic parameters (Pattern, SR, SH). The overall success rate for the realization of elastographic examination was 90%. In five cases, the elastography failed due to obesity [patients’ body mass index (BMI) was 30,02 – 36,8 kg/m^2^]. Of the mentioned five cases, infiltration of 1 sentinel LN by macro metastasis was histologically confirmed in 2 patients. **Figures 3** and **4** present elastography images comparing metastatic lymph nodes with benign ones during surgery.

### 3.5 B-mode USG findings

**Figure 5:**
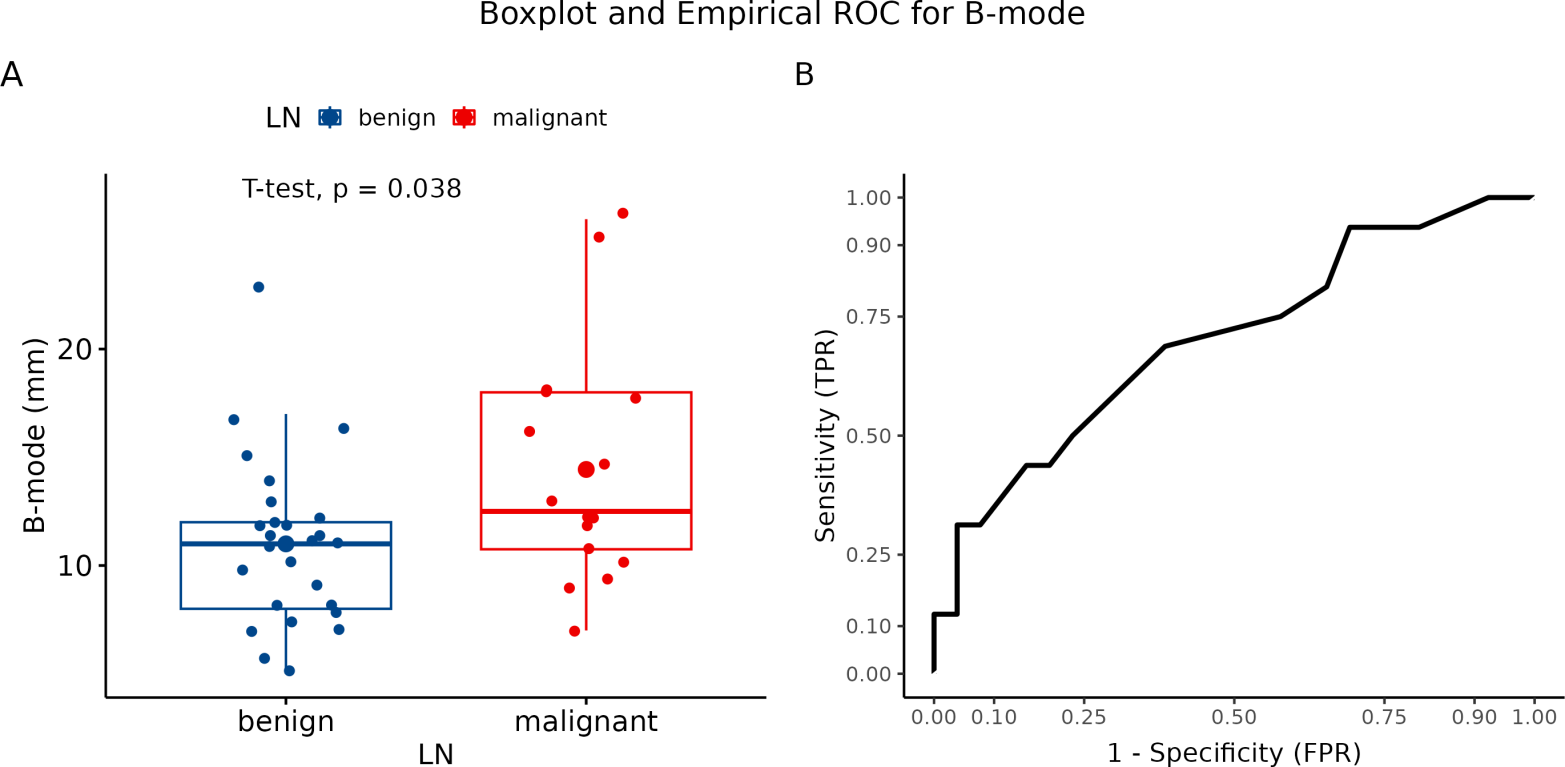
Evaluation using B-mode ultrasonography. **(A)** Boxplot and swarmplot of data points for B-mode. Benign cases are represented in blue, while malignant cases are shown in red. The larger dot indicates the mean value for each group. The p-value is derived from a two-sample t-test. **(B)** Empirical Receiver Operating Characteristic (ROC) curve for B-mode. In this plot, TPR refers to the True Positive Rate, and FPR denotes the False Positive Rate. LN, lymph node.

The B-mode USG method was used to evaluate signs of malignant transformation of LNs (spherical LNs, change in echogenicity, and signs of a ruptured capsule). We analyzed number of observed parameters. Afterward, we compared the results of the B-mode USG observation to histological findings of LNs status. Further, we determined the sensitivity and specificity of B-mode in differentiating malignant LNs from benign ones. The frequency of USG signs of LNs involvement by metastases in histologically verified benign and metastatic LN is graphically summarized using a box plot (**Figure 5A**). The association between LN without and with metastasis was statistically significant (p = 0.038). Using the Youden Index of USG at B-mode to differentiate the LN state, we estimated a sensitivity and specificity of 68.75% and 61.54%, respectively (**Figure 5B**).

### 3.6 Qualitative elastographic findings (pattern)

**Figure 6:**
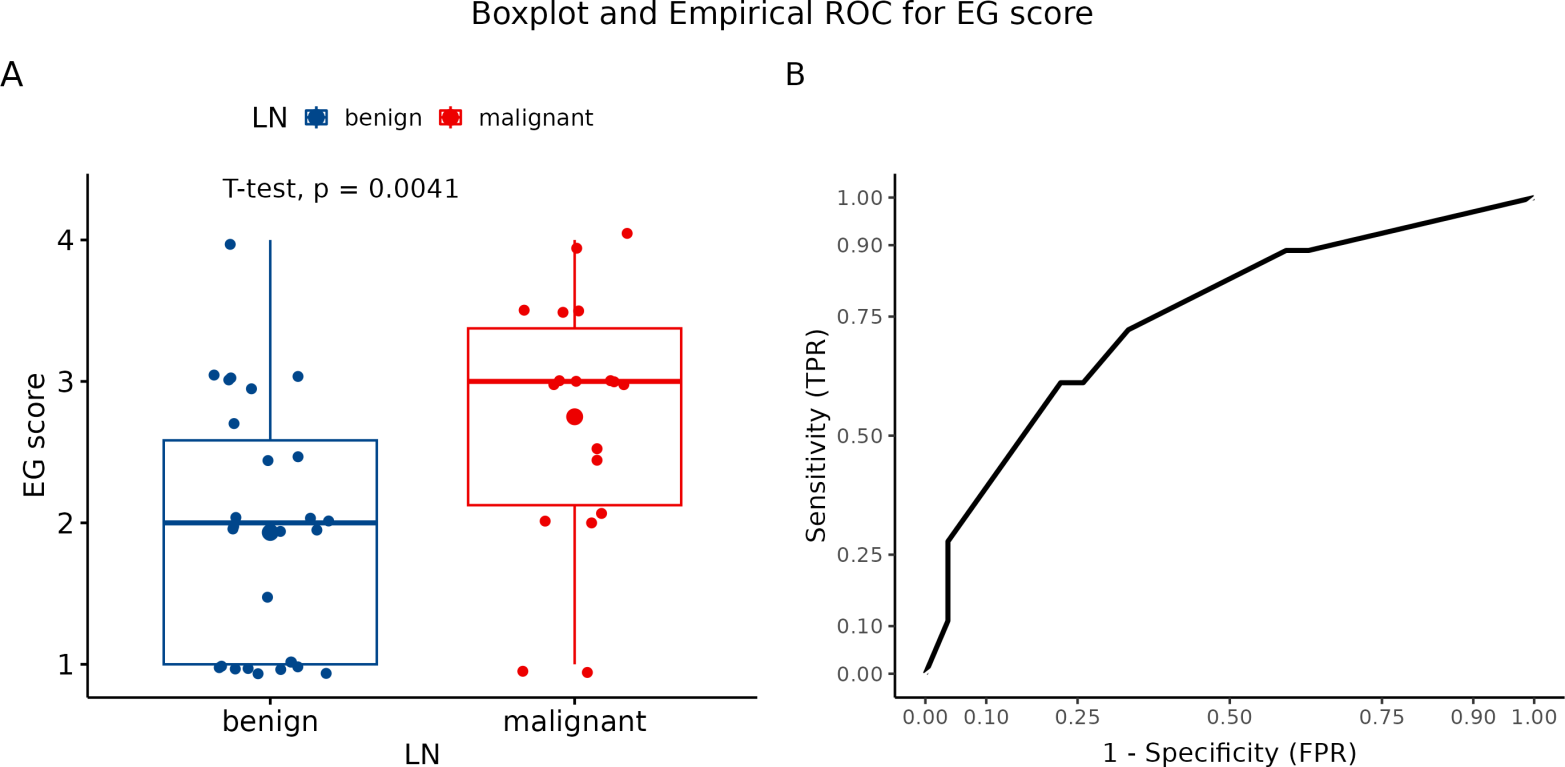
**(A)** Boxplot and swarmplot of data points for EG score. Benign cases are represented in blue, while malignant cases are shown in red. The larger dot indicates the mean value for each group. The p-value is derived from a two-sample t-test. **(B)** Empirical Receiver Operating Characteristic (ROC) curve for EG score. In this plot, TPR refers to the True Positive Rate, and FPR denotes the False Positive Rate. LN, lymph node.

In the study design, we planned to evaluate three LNs of each patient. Overall, we performed only three examinations of all three LNs out of 45 patients who underwent elastography. Two LNs were examined in 26 patients. The four-point elastographic scale was used for the evaluation of elastographic patterns. Elastography patterns I and II were classified as benign and III and IV as malignant. Of 45 cases, a benign pattern was estimated in 21 patients (P I and P II), and a malignant pattern (P II and P IV) was identified in 24 patients. In 18 cases, the classification of LNs by pattern showed the benign character of tissue correctly, while in 15 cases, LNs were evaluated as benign. According to our results, the color pattern showed a diagnostic accuracy of 73.3%. The number of clinically relevant false negative and clinically irrelevant false positive results was 3 (6.67%) and 9 (20%), respectively. The sensitivity and specificity of this parameter were 83.3% and 66.67%, respectively (**Figure 6A**). In addition, PPV was 62.5% while NPV was 85.7%. For graphical summarization of individual patterns (EG score) depending on the dignity of LNs (histologically classified), we set up a median for benign LN (P II) and malignant LN (P III) (**Figure 6B**).

### 3.7 Quantitative elastographic findings (strain ratio)

**Figure 7:**
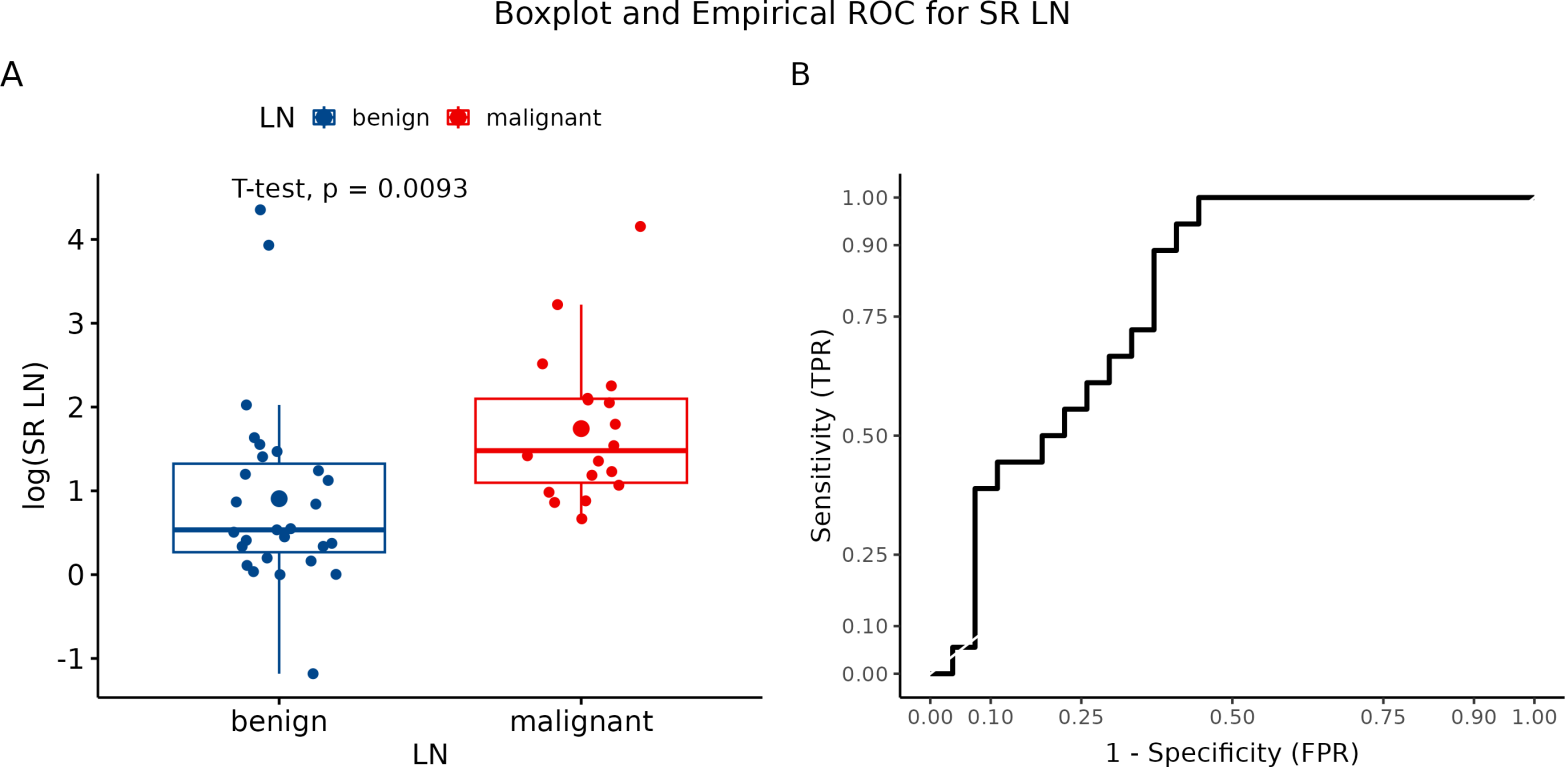
**(A)** Boxplot and swarmplot of data points for SR-LN. Benign cases are represented in blue, while malignant cases are shown in red. The larger dot indicates the mean value for each group. The p-value is derived from a two-sample t-test. **(B)** Empirical Receiver Operating Characteristic (ROC) curve for SR-LN. In this plot, TPR refers to the True Positive Rate, and FPR denotes the False Positive Rate. LN, lymph node.

Using semiquantitative strain ratio (SR), we performed three measurements for each visualized LN to eliminate methodology errors. Axillary fat (fat-to-lesion ratio) was used as a reference tissue for LNs evaluation. The average SR value was 6.98 for benign LN and 9.58 for metastatic LN. Logarithmic transformation to realize a more normal distribution revealed significant differences between malignant and benign LN (p = 0.0093) (**Figure 7A**). In our study, we identified SR with a sensitivity of 100% and a specificity of 55.6% (**Figure 7B**).

### 3.8 Quantitative elastographic findings (hue elastogram – elasticity score)

**Figure 8:**
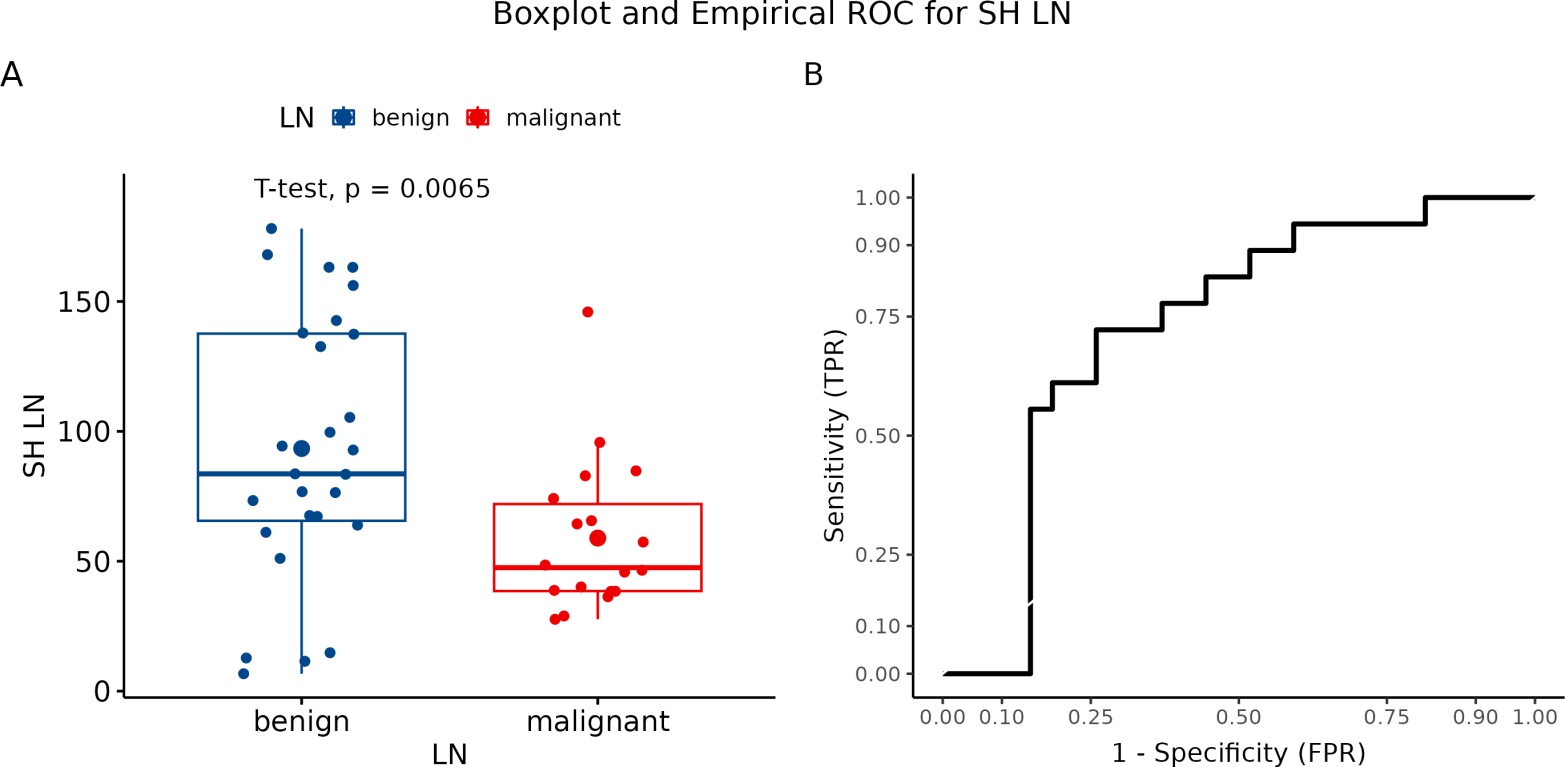
**(A)** Boxplot and swarmplot of data points for SH-LN. Benign cases are represented in blue, while malignant cases are shown in red. The larger dot indicates the mean value for each group. The p-value is derived from a two-sample t-test. **(B)** Empirical Receiver Operating Characteristic (ROC) curve for SH-LN. In this plot, TPR refers to the True Positive Rate, and FPR denotes the False Positive Rate. LN, lymph node.

We evaluated each visualized LN in three measurements. Subsequently, we calculated the mean of all three values, which were further statistically processed. The final mean value for benign LN was 93.39, while metastatic LN showed a mean value of 58.915 (p = 0.0065) (**Figure 8A**). Hue histogram showed a sensitivity of 72.2% but a specificity of only 25.9% (**Figure 8B**).

### 3.9 Comparison of B-mode and B-mode + elastography

**Figure 9:**
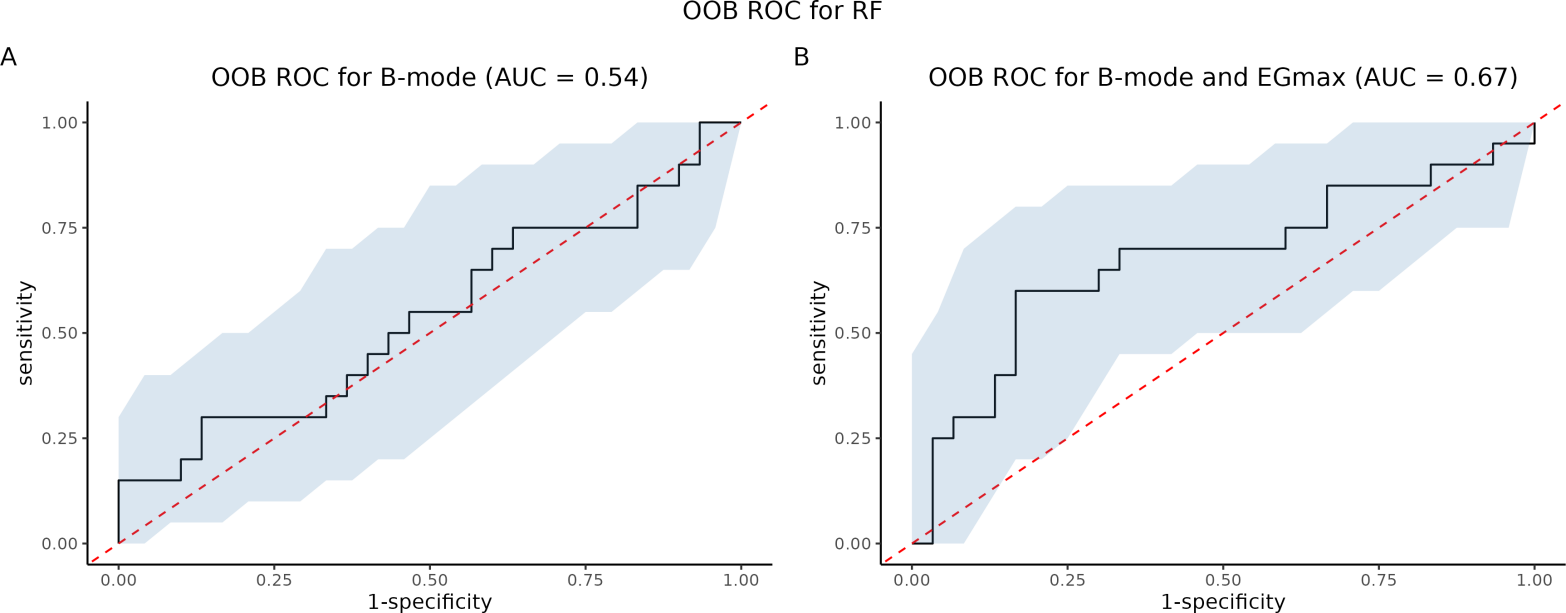
Comparison of B-mode and elastography. OOB ROC curves for **(A)** B-mode and **(B)** Bmode+EGmax.

We compared strain elastography (SE) to B-mode ultrasound. We trained two Random Forest (RF) models: one using B-mode ultrasound as the sole predictor (RF-B), and another using both B-mode ultrasound and EGmax as predictors (RF-B-E). Then, we constructed Out-Of-Bag (OOB) ROC curves and compared the corresponding AUCs using both Venkatraman’s test and DeLong’s test. Then, we tested the null hypothesis of equality of AUCs, in paired setting. The AUC for RF with B-mode was 0.54, for RF with both B-mode and EGmax it was 0.67. P-value from Venkatraman’s test was 0.0075, whereas Delong’s test led p-value 0.3827. All comparisons are summarized in **Figure 9**. **Table 3** summarized performance metrics (sensitivity, specificity, NPV, PPV, accuracy, precision, recall, F-measure, and Youden index) for both Random Forest models (RF-B and RF-B-E) based on the OOB class predictions.

**Table 3:** Detailed performance metrics for both for both Random Forest models (RF-B and RF-B-E) based on the OOB class predictions.

Performance Metrics for RF-B (B-mode only)		Performance Metrics for RF-B-E (B-mode + EGmax)	
Metric	Value	Metric	Value
Sensitivity	0.50	Sensitivity	0.70
Specificity	0.45	Specificity	0.65
NPV	0.38	NPV	0.59
PPV	0.58	PPV	0.75
Accuracy	0.48	Accuracy	0.68
Precision	0.58	Precision	0.75
Recall	0.50	Recall	0.70
F-measure	0.54	F-measure	0.72
Youden Index	-0.05	Youden Index	0.35

NPV, negative predictive value; PPV, positive predictive value.

**Figure 10:**
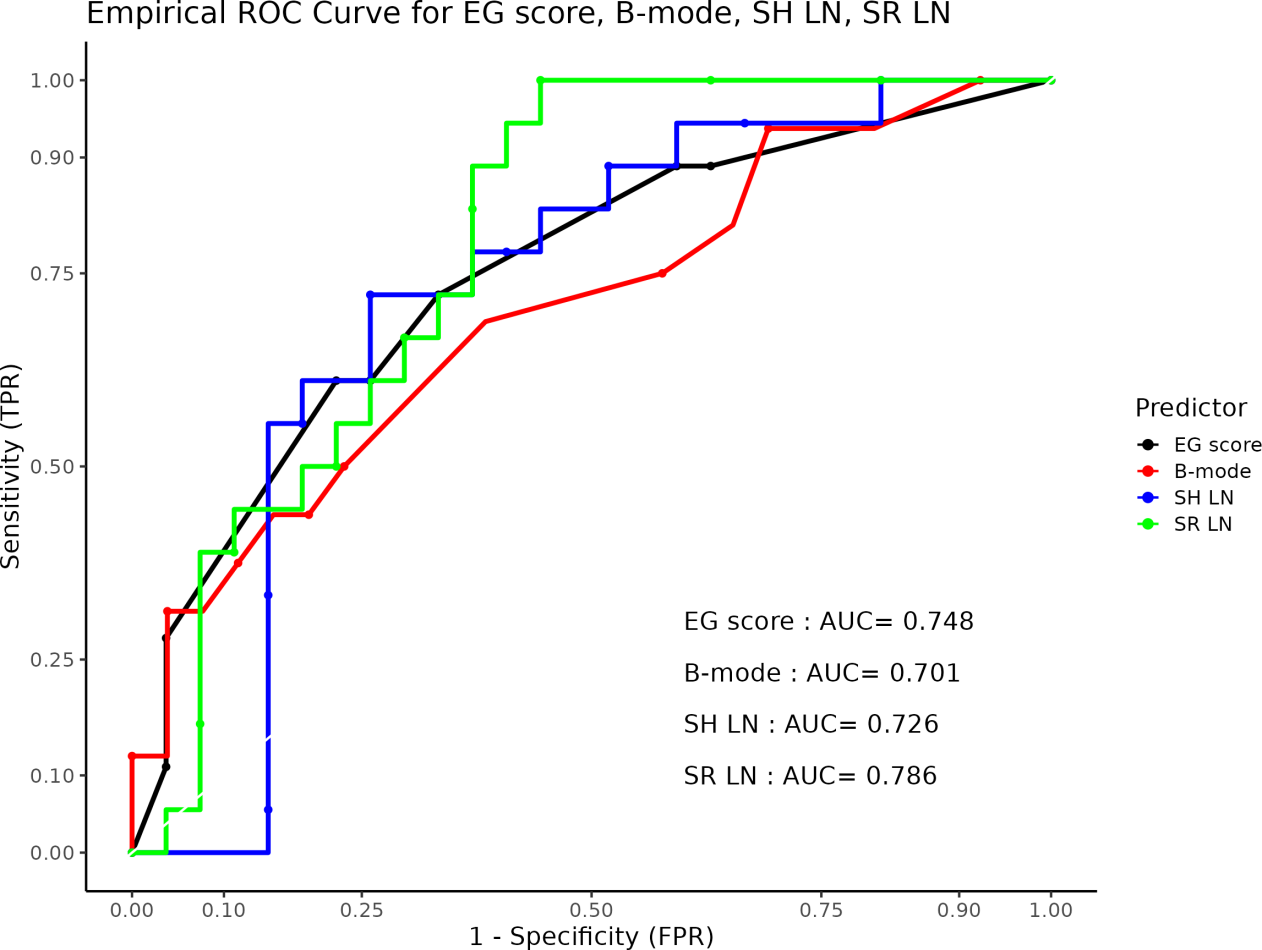
Empirical Receiver Operating Characteristic (ROC) curve for EG score (black), B-mode (red), SH LN (blue) and SR LN (green). In this plot, TPR refers to the True Positive Rate, and FPR denotes the False Positive Rate, AUC to the Area Under ROC curve. LN, lymph node.

A comparison of all three parameters of elastography is summarized in **Figure 10**. Based on acquired data, the best results showed pattern (EG score) and strain ratio. Using ultrasonographic B-mode, we revealed lower sensitivity. Finally, the hue histogram exerted good sensitivity but low specificity.

## 4 Discussion

The differentiation between malignant and benign tumors using a non-invasive method represents an innovative technique to achieve improved characterization of tumor and patient-tailored therapy. Currently, histopathological examination of tumor tissue obtained from biopsy remains the gold standard for preoperative BC diagnosis (18). Real-time elastography is a relatively novel, promising, non-invasive method to reveal the viscoelastic properties of tissue to generate qualitative and quantitative assessment of elasticity values, developed as an alternative to tissue biopsy (19).The aim of the study was to investigate a non-invasive elastographic imaging technique for the assessment of LN status in the cohort of patients with early-stage BC (T1 and T2). In addition, according to the heterogeneity of stiffness, we attempted to distinguish benign and malignant LN and compared acquired results from elastography examination with routinely used B-mode USG and physical examination in clinical staging of axillary LNs in BC patients. B-mode USG represents one of the recommended imaging techniques for the determination of node staging in gastric, pancreatic, and esophageal cancer (20–22). Above all, previous studies have found that the specificity of conventional B-mode imaging is not high (around 80%) (23, 24). In our study, the B-mode USG examination yielded lower specificity compared to previously mentioned studies (only about 60%). As was reported in several studies, elastography-based imaging techniques can distinguish malignant from benign masses by their stiffness (25–27). Giovannini et al. (2006) used sonoelastography to differentiate malignant and benign pancreatic tissue and LNs. The elastographic examination for LN invasion revealed a sensitivity of 100%, while a specificity was only 50% (28). In another study, Janssen et al. (2008) confirmed the accuracy of endosonographic elastography at 85% - 90% for differentiation between malignant and benign LNs (23). The benefits of ultrasound elastography include real-time visualization, non-invasiveness, and high specificity that can be effectively used in the differentiated diagnostic of malignant diseases and determination of LN involvement (29). It is important to note that the false negative rate of sentinel LN biopsy of up to 10% is defined as non-acceptable (corresponding to a sensitivity of 90%) (30). Therefore, innovative non-invasive methods to determine the malignant status of LNs can be considered accurate and reliable only in accordance with acceptation of a mentioned criteria.

The diagnostic performance of ultrasound elastography provides two types of evaluation, quantitative (strain ratio and hue histogram) and qualitative (color pattern) (31). Up to now, several studies have evaluated elastography for the determination of axillary staging (all observations were confirmed by histopathology analysis). The authors of these studies distinguished malignant from benign tissue with a specificity of 56.2 – 95.65% and a sensitivity of 66.7% – 86% using qualitative elastographic patterns (27, 32–36). In our study, we identified a specificity of 66.67% and a sensitivity of 83.3% by the use of elasticity patterns. Only Onol et al. (2020) reported results in which the sensitivity of elastography was higher than 90% (36). The American Society of Clinical Oncology (ASCO) analyzed false negative rates for sentinel LNs detected by the most common technique (radionuclides, patent blue) based on the results from 6 studies in the range of 4.6 – 16.7% (average of 8.4%). The American Society of Breast Surgeons (ASBrS) defined the acceptable tolerance limit of false negative rates for sentinel LN detection as 5% (37). In 2018, Xu et al. reported false negative rates of 13.5% (35). Our findings revealed false negative results in 3 cases (false negative rates 6.67%). On the other hand, false-positive results were documented in 9 cases (20%). The false positive rates do not include risk for patients because all probands were indicated for sentinel LN biopsy.

As a semiquantitative parameter, SR provides information by comparing a lesion to the surrounding normal tissue (38). This parameter correlates with malignancy or benignity characteristics of lesions in which a higher SR ratio is generally considered for malignancy while a lower SR ratio indicates a benign lesion. Doaa et al. (2016) showed SR ratio of 1.60 for benign LNs and 3.45 indicating malignancy (39). In another study, Xu et al. (2018) reported SR value of 2.35 *±* 1.80 for benign and 12.64 ± 5.30 for malignant LNs (35). In the Okasha et al. study, the SR ratio was > 4.6 for malignant masses (40). Our results showed an average SR ratio for benign LNs of 6.98 and lesions with SR of 9.58 were categorized as malignant. The clinical studies using real-time strain elastography reported an overall sensitivity of 71.6% (34), 85% (41), 87% (35), 93.3% (39), and 100%, while a specificity was in the range of 75 – 76% (34, 35, 39). Higher specificity (98%) was documented in a study by Lyshchik et al. (2007) (41). Our results are comparable to that of Pehlivan et al. (42) Thus, we confirmed 100% sensitivity and only 55.6% specificity of strain elastography. The use of SR parameter of elastography can be an adjunctive tool to ultrasonography, improving accuracy from 70.8% (USG) to 84.3% (elastography) (43). Novel ultrasound platforms have integrated software for the evaluation of hue histogram. A Hue histogram is generated by converting color frames to numerical form to obtain the elasticity information (44). Nowadays, hue histogram analysis of real-time elastography is used for the non-invasive assessment of liver diseases and along with endosonography for the assessment of pancreatic tumors (45). Evaluating LN metastasis *via* hue histogram analysis is currently practiced in veterinary medicine. In a study by Choi et al. (2019) focusing on the differentiation between non-metastatic and metastatic LNs in dogs, a sensitivity and specificity 100% and 92%, respectively, were obtained (46). Our study showed that although hue histogram analysis demonstrated 72.2% sensitivity, the specificity was only 25.9%. Currently, most cases of breast carcinoma are diagnosed in the early stage, without spreading to the axillary LNs. Preoperative diagnosis of LNs includes fine needle aspiration (FNA) biopsy and USG with an overall diagnostic sensitivity of 80 – 100%. The mentioned level of sensitivity is directly proportional to the size of the affected LN. For small LNs (up to 5 mm in size), the sensitivity of FNA and USG decreases to around 44% (47). This limitation can be eliminated by combining elastography and FNA biopsy, allowing precise identification of the part of the LN with the highest hardness, thus allowing LN biopsy to be more precise. Therefore, combining elastography and USG can represent one of the most effective ways to reduce false positive results in the preoperative management of LN status (44). Moreover, nonsurgical (clinical) axillary LNs assessment shows high false positive results rates. Subsequent histological analysis reveals up to 70% clinical positive LNs without metastatic involvement (48). In our study, we observed low sensitivity (50%) of clinical examination (a specificity was 96.7%). Therefore, the assessment of axillary LNs by palpation is demonstrated to be inaccurate, resulting in the overmedication of patients and increased risk of lymphoedema development or other health complications as a result of the low sensitivity of clinical examination.

Also, it is important to acknowledge several limitations in our study. Briefly, it can be summarized in these points: 1) the variability in the results of available studies and small sample sizes, which are significantly biased towards malignant LNs, arise from the clinical necessity of their histological analysis; 2) lack of standardization of the strain elastography method; 3) some malignant LNs are not significantly stiffer (e.g., in malignant lymphoma, LNs have similar elasticity to reference tissue).

In conclusion, according to our observation and results from similar published studies focused on the elastography examination of LNs status, strain elastography is currently inappropriate for replacing invasive sentinel LN biopsy. However, as the adjuvant diagnostic tool, strain elastography showed usefulness for improving the evaluation capacity of USG and allows more precise FNA biopsy.

## Data Availability

The raw data supporting the conclusions of this article will be made available by the authors, without undue reservation.
